# Type1 Interferons Potential Initiating Factors Linking Skin Wounds With Psoriasis Pathogenesis

**DOI:** 10.3389/fimmu.2019.01440

**Published:** 2019-06-25

**Authors:** Ling-juan Zhang

**Affiliations:** ^1^School of Pharmaceutical Sciences, Xiamen University, Xiamen, China; ^2^Department of Dermatology, University of California, San Diego, La Jolla, CA, United States

**Keywords:** type 1 interferons, interferon beta, innate immunity, keratinocytes, inflammation, skin wounds, psoriasis

## Abstract

Psoriasis is a chronic autoimmune skin disease that can often be triggered upon skin injury, known as Koebner phenomenon. Type 1 interferons (IFNα and IFNβ), key cytokines that activate autoimmunity during viral infection, have been suggested to play an indispensable role in initiating psoriasis during skin injury. Type 1 IFN-inducible gene signature has been identified as one of the major upregulated gene signatures in psoriatic skin. Type 1 IFNs treatments often directly induce or exacerbate psoriasis, whereas blocking type 1 IFNs signaling pathway in animal models effectively inhibits the development of T cell-mediated skin inflammation and psoriasis-like inflammatory diseases. Epidermal keratinocytes (KCs) occupy the outermost position in the skin and are the first responder to skin injury. Skin injury rapidly induces IFNβ from KCs and IFNα from dermal plasmacytoid dendritic cells (pDCs) through distinct mechanisms. Host antimicrobial peptide LL37 potentiates double-stranded RNA (dsRNA) immune pathways in keratinocytes and single-stranded RNA or DNA pathways in pDCs, leading to production of distinct type 1 IFN genes. IFNβ from KC promotes dendritic cell maturation and the subsequent T cell proliferation, contributing to autoimmune activation during skin injury and psoriasis pathogenesis. Accumulating evidences have indicated an important role of this dsRNA immune pathway in psoriasis pathogenesis. Together, this review describes how skin injury induces type 1 IFNs from skin cells and how this may initiate autoimmune cascades that trigger psoriasis. Targeting keratinocytes or type 1 IFNs in combination with T cell therapy may result in more sustainable effect to treat auto-inflammatory skin diseases such as psoriasis.

## Introduction

Skin, the largest organ of human body, functions as a physical and immunological barrier to protect our bodies from external threats. The epidermis, positioned at the front line of defense, has evolved to provide rapid and specific innate immune response that shapes the adaptive immune response, leading to immediate as well as long term protection against physical dangers (1, 2). The barrier function of epidermis is primarily provided by keratinocytes (KCs), the pre-dominant epidermal cell type. While the physical barrier of the skin is maintained by a tightly controlled balance between proliferation and differentiation of KCs (3, 4), the immunological barrier function of epidermis relies on rapid, precise and situation-specific innate immune responses of KCs to insults. Psoriasis is considered as a T cell-mediated autoimmune skin disease, whereas the role of KCs in initiating the early upstream events in psoriasis has been under-appreciated. In this review, we review current understanding of immunopathology of psoriasis, the role of keratinocytes in psoriasis initiation, and then emphasize on the role of type 1 IFNs in linking innate immune activation upon skin injury and the subsequent autoimmune amplification that leads to psoriasis.

## Psoriasis: Etiology, Immunopathogenesis and Therapies

Psoriasis is a chronic autoimmune skin disorder characterized by well-demarcated, raised areas of erythematous plaques, often covered by silvery scaling (5). It is estimated that psoriasis affects 125 million people worldwide (~1.7% of the world population), including 2~4% of the US or European populations and ~0.5% of the Asians (6). The three principal histological features of psoriasis are hyperplastic/thickened epidermis, elongated and increased vascularity in the dermis, and inflammatory leukocyte infiltration (5). Beyond the lesions, psoriasis is also associated with several comorbidities, such as rheumatoid arthritis, atherosclerosis, cardiovascular diseases, obesity, type 2 diabetics, Alzheimer's disease, depression, and non-melanoma skin cancer (7, 8). Increased disease burden greatly impairs the quality of life in psoriasis patients. A better understanding of psoriasis pathophysiology will help to decipher the molecular alliance of psoriasis with its comorbidities.

**Figure 1:**
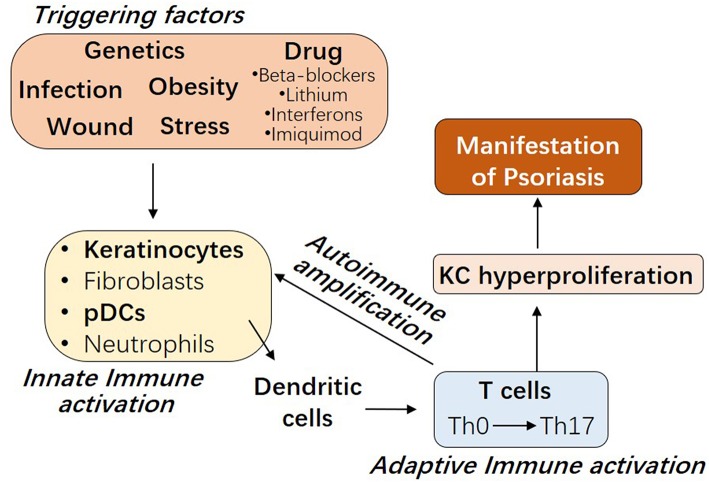
The interplay between innate and adaptive immune cells in the pathogenesis of Psoriasis. Psoriasis can be triggered by several factors, including genetic variants, infection, wound, obesity, stress, and drugs. Early upstream events in psoriasis involve the innate immune activation of skin resident keratinocytes or fibroblasts or recruited plasmacytoid dendritic cells (pDCs) or neutrophils. Cytokines derived from these innate immune cells promote myeloid dendritic cell maturation, with consequent Th17 T cell development and the beginning of the adaptive immune phase. T cell infiltrate promotes inflammatory amplification of innate immune cells, leading to the formation of an autoimmune self-amplifying loop that drives pathogenic hyperproliferation of keratinocytes and manifestations of psoriasis.

The etiology of psoriasis remains obscure. As shown in Figure 1, besides genetic factors, several triggering factors have been linked with an exacerbation of psoriasis, such as infection, wound, obesity, stress, and drugs including beta-blockers, lithium, interferons, and imiquimod (9–11). Innate immune responses of resident keratinocytes or infiltrated plasmacytoid dendritic cells (pDCs) are believed to play a critical role in initiating the subsequent adaptive immune events including dendritic cell (DC) maturation and T cell activation (2, 12, 13). It has been shown that keratinocyte or pDCs derived cytokines, such as IL1β and type 1 IFNs, activate DC, which then stay local or travel to lymph nodes and secrete cytokines TNFα, interleukin (IL)-12 and IL-23, leading to type 1 T helper (Th1), and type 17 T helper (Th17) cell activation, respectively (2, 14). Activated T cells accumulate at the affected skin area, releasing additional cytokines, such as IFNγ, TNFα, IL-22, and IL-17A (15). These T cell derived cytokines recruit additional immune cells and boost keratinocyte activation, leading to the initiation of a self-propelled cycle of auto-inflammation and the ultimately uncontrolled keratinocyte hyperproliferation and psoriatic plaque formation (16, 17).

Preventing immune activation is the key to treat psoriasis. Conventional psoriasis therapies, including general immunosuppressive therapies (such as topical corticosteroids and systemic methotrexate or cyclosporin therapies), vitamin D analogs, topical retinoids, and UVB phototherapy, are associated with broadband immunosuppression and/or organ toxicities that can be problematic when used long term (18). New biological drugs against specific immunological elements have gained popularity as safe and effective alternatives to treat moderate to severe plaque psoriasis. These biological drugs including monoclonal antibodies against TNFα (such as etanercept and infliximab), IL12 (such as Ustekinumab), IL23 (such as Guselkumab and Tildrakizumab), IL-17A (such as Secukinumab and Ixekizumab), or IL17AR (such as Brodalumab) have shown clinical efficacy in improving skin conditions in clinical trials and most of these drug are already on the market (9, 19–21). However, relapse of the disease shortly after drug withdrawal is one of the major obstacles for these DC or T-cell targeted therapies in clinical trials (22–25), suggesting that preventing adaptive immune activation alone is not sufficient to treat psoriasis. A better understanding of the innate immune mechanisms initiating psoriasis is urgently needed to develop novel therapeutic approach to treat psoriasis.

## Koebner Phenomenon, From Skin Wound to Psoriasis

The initial onset of psoriasis is often followed by chronic relapses of the disease triggered by wounding, infections and mechanical stress (5). In 1876, Hinrich Koebner, MD, first described the development of psoriatic lesion after physical trauma such as tattoos, horse bites, and wounds. Later, “Koebner phenomenon” has been used to describe the formation of isomorphic lesions on healthy skin following a cutaneous trauma (e.g., wounds, burns, or surgical incisions) and it is now not restricted to psoriasis but applies to many other skin conditions, such as lichen planus, vitiligo, and lupus erythematosus. Dermal pDCs, a rare population of circulating cells specialized in the production of type 1 IFNs, is thought to be one of the earliest events in psoriasis pathogenesis and subsequently primes the innate and adaptive immune system (12, 26). While dermal pDC activation in wounded skin may partially explain Koebner phenomenon, it is still unclear why even superficial tattoos can trigger the pathogenesis of psoriasis. An epidermal mechanism is likely to play a role in the development of a Koebner reaction in psoriatic patient.

## Keratinocytes Under Fire of Proinflammatory Cytokines

Keratinocytes, constituting ~90% of the epidermal cells, are poised directly at the interface with the external environment, depositing them as the first responder to skin injury. Recent studies have established the essential role of keratinocytes in psoriasis initiation. Rapid innate immune responses of keratinocytes, to a variety of external stimuli, leads to production of an array of pro-inflammatory cytokines or chemokines such as IFNβ, IL1β, IL36, TNF, IL6, IL8, IL25, and CXCL10 (2, 27–29). These keratinocyte-derived cytokines prime epidermal innate immune signals with dermal adaptive immune system, contributing to autoimmune activation and psoriasis pathogenesis (2, 3, 27, 28, 30, 31).

The inflammatory T cell phenotype of psoriasis can be initiated by altering innate immune system of keratinocytes in mice. For example, epidermal specific deletion of IKK2 (inhibitor of nuclear factor κB) (32) or c-JUN (33) or epidermal specific overexpression of Tie2 (34) or IL17C (35) or the active form of STAT3 (36) or IL25 (29) lead to spontaneous keratinocyte activation and cytokine release followed by the development of psoriasis-like skin inflammation. Recent study from Kabashima's group shows that conditional deletion of TRAF6 in keratinocytes abrogates DC activation, IL-23 production, and the subsequent IL-17 mediated psoriatic inflammation in the imiquimod psoriasis mouse model (37). Our group have found that induction of IFNβ from KC is one of the earliest innate immune events during skin injury (2). Keratinocyte-derived IFNβ promotes dendritic cell maturation and the subsequent T cell proliferation, leading to psoriatic inflammation development (2). These studies suggest that innate immune responses of keratinocytes are essential to initiate the autoimmune cascade and drive psoriasis pathogenesis, and type 1 IFNs may function as an early initiating factor linking skin wounds with adaptive immune activation that drives psoriasis.

## Type 1 IFNs and Autoimmune Diseases

Type 1 interferons (IFNs) belong to the class II family of cytokines, which is composed of 16 members, including 13 IFNα subtypes, IFNβ, IFNε, IFNκ, and IFNω (38). Among these type 1 IFNs, IFNα, and IFNβ are the most extensively studied. Type1 IFNs were first discovered more than 60 years ago as the key factors induced upon viral infection, owing to their ability to limit viral replication and promote immune activation (39–41). And now type 1 IFNs have been recognized as the central cytokines that link innate immunity with autoimmune activation during pathogenesis of several systemic autoimmune diseases and several organ-targeted inflammatory diseases (42, 43). Systemic lupus erythematosus (SLE) is the most well-studied autoimmune disease driven by type1 IFNs (42), and recent studies have also demonstrated pathogenic role of type 1 IFN in psoriasis, rheumatoid arthritis, diabetes mellitus, Sjogrens syndrome, dermatomyositis (DM), and systemic sclerosis (38, 44–46). SLE and psoriasis share many similar clinical features and can both be triggered by infection, wounding or type1 IFNs, and Th17 T cells contribute to pathogenesis of both diseases. However, SLE is characterized by overproduction of a wide array of autoantibodies and is therefore traditionally classified as a “B-cell disease” (47), whereas B cell contribution to psoriasis remains unclear. Distinct susceptible genes have been identified for SLE and psoriasis (6, 48), suggesting that genetic factors may contribute differential adaptive immune development in response to type 1 IFNs, leading to distinct disease manifestation in SLE and psoriasis.

## Type 1 IFNs and Psoriasis Pathogenesis

Type 1 IFNs are associated with psoriasis. Psoriasis is often triggered by chronic viral infections or wounding, and type 1 IFNs as the key cytokines induced upon these conditions (49, 50). Clinically, type 1 IFNs treatments in patients with viral infection or multiple sclerosis (MS) often directly induce or exacerbate psoriasis or psoriasis arthritis (50–53). Furthermore, transcriptome analyses have identified type 1 IFN pathway genes as one of the top upregulated gene signatures in psoriatic skin, outscoring the upregulation of TNFα pathway gene signature (54–56). Immunostaining analyses have shown that while IFNα is pre-dominantly produced by dermal infiltrated pDCs, IFNβ expression is rapidly induced in epidermal keratinocytes as early as 1 day post-wounding and in psoriatic epidermis compared to normal human skin (2, 12, 13).

In line with these clinical observations, type 1 IFN pathway is also necessary for the development of T cell-mediated skin inflammation and psoriasis-like inflammatory diseases in mice. Mice treated with IFNα or IFNβ neutralizing antibodies, or mice lacking IFNAR (receptor for IFNα/β) failed to develop Th17 cell-mediated skin inflammation (12, 13). Fuchs's group shows that UV alleviates the imiquimod (IMQ)-induced psoriatic inflammation by downregulating IFNAR1 expression in keratinocytes (57). IMQ-induced psoriatic inflammation was blocked in *Ifnar* deficient (*Ifna*r^−/−^) mice, and in contrast *Ifna*r^*SA*^ mice (in which *Ifnar* ubiquitination and degradation was blocked) exhibited exacerbated inflammatory response to IMQ compared to wildtype controls (57), demonstrating that IFNAR plays a critical role in promoting the development of psoriasis like inflammation in mice. In addition, mice deficient for interferon regulatory factor 2 (*Irf 2*), the transcriptional attenuator of IFNα/β signaling, developed spontaneous psoriasis-like inflammatory disease (58). Type 1 IFNs upregulates the expression of IL22 receptor in keratinocytes, leading to an increase in keratinocytes' responsiveness to IL22, which drives Stat3 phosphorylation and keratinocyte hyperproliferation (15). Our group has also shown that IFNβ from activated human or mouse keratinocytes can directly promote cDC (conventional dendritic cell) maturation and the subsequent T cell proliferation *in vitro* (2). These collective evidences support that type 1 IFNs play an essential role in initiating skin inflammation during psoriasis pathogenesis.

In addition to its proinflammatory role, type 1 IFNs may also regulate keratinocyte differentiation. In both normal and psoriatic skin epidermis, IFNβ expression is restricted to KCs at the differentiated cell layers (2, 59, 60), and IFNβ is expressed by growth arrested or differentiated KCs but not by dividing KCs *in vitro* (59). Neutralizing IFNβ in culture medium inhibited differentiation, but addition of exogenous IFNβ did not stimulate differentiation or alter proliferation (59). Future studies are still needed to determine whether IFNβ expression in differentiated KCs is a consequence or the cause of KC cell arrest and/or differentiation, and to determine whether IFNβ contributes to the aberrant proliferation and differentiation in psoriasis.

## Distinct Cellular Source of IFNα and IFNβ in Psoriasis

**Figure 2:**
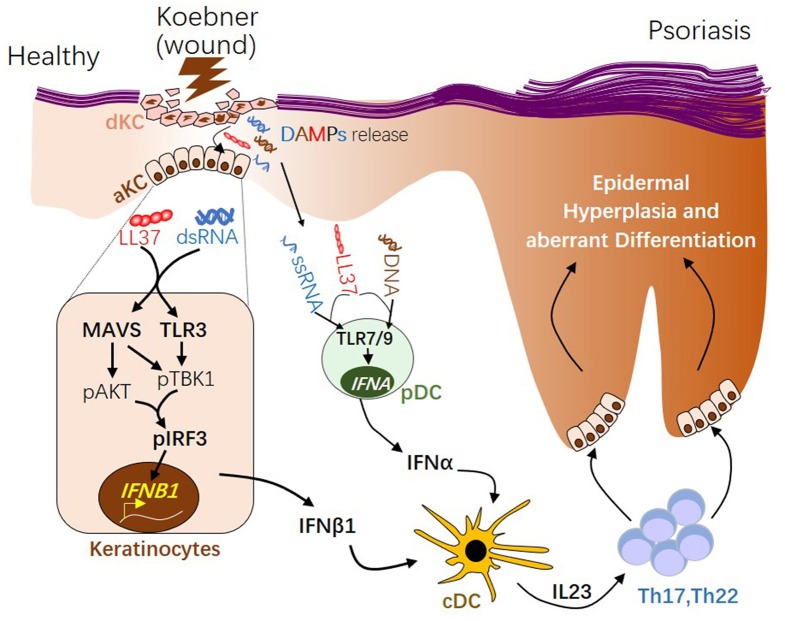
The role of type 1 interferons in initiating psoriatic inflammation during skin injury. During skin injury (known as Koebner phenomenon), damaged keratinocytes (dKC) release self-nucleic acids, including dsRNA, ssRNA, and DNA. Antimicrobial peptide LL37, which is transiently induced in KC upon wounding enables dsRNA recognition by MAVS and TLR3 in KC, leading to activation of the pAKT-pTBK1-pIRF3 signaling cascade and the subsequent transcription initiation of the *IFNB1* gene from activated KCs (aKC). LL37 can also enables ssRNA or DNA recognition by TLR7 or 9 in pDCs, leading to transcription of *IFNA* family genes. Type 1 IFNs, including IFNβ from KC and IFNα from pDCs, promote maturation of conventional DCs (cDCs). Activated DCs produce IL23, promoting the development of Th17 and Th22 autoimmune T cells, which in turn act on keratinocytes, leading to epidermal hyperplasia and psoriasis pathogenesis. dsRNA, double-stranded RNA; ssRNA, single-stranded RNA; MAVS, Mitochondrial Antiviral Signaling Protein; TBK1, TANK-Binding Kinase 1; IRF3, interferon regulatory factor 3.

IFNα and IFNβ are produced by distinct cellular sources in wounded and/or psoriatic skin. Upon skin injury, pDCs rapidly infiltrate the skin, sense nucleic acid released from damaged cells, then produce large quantity of IFNα, which then initiates the autoimmune cascade (12, 13) (Figure 2). Activation of pDC precedes cDC or T cells activation (12), suggesting IFNα from pDC may play a role during early phase of disease progression. Our group has shown that while IFNα is primarily produced by pDC in the dermis, IFNβ is pre-dominantly produced by epidermal keratinocytes in skin wounds or psoriasis (2). Direct comparison of *in vitro* activated KCs and pDCs revealed that while KCs lack the ability to produce IFNα upon activation, activated KCs produce higher amount of IFNβ compared to activated pDCs (2). Secretion of IFNβ from keratinocytes promotes activation and maturation of classical dendritic cells, leading to the subsequent T cell proliferation and autoimmune amplification (2). Furthermore, keratinocyte-derived IFNβ can also promote pDC maturation and activation (2), suggesting that keratinocytes might also contribute to pDC activation during early phase of skin injury. Together, these findings suggest that KCs are an active source of IFNβ and can participate with pDCs to prime the adaptive immune system during psoriasis pathogenesis (Figure 2).

## Regulation of Type1 IFNs Expression by Pattern Recognition Receptors

Type 1 IFNs are known to be induced by a variety of DAMPs (damage associated molecular patterns) or PAMPs (pathogen associated molecular patterns) in either through Toll-like receptor (TLR)-dependent or TLR-independent pathways (38, 61) in a cell type specific manner. Type1 IFNs can be induced upon activation of endosomal TLR7 and 9 or cytosolic cGAS-STING (cyclic GMP-AMP synthase-stimulator of interferon genes) by host or viral or bacterial DNA, endosomal TLR8 by ssRNA, endosomal TLR3 or mitochondrial RIG1 (retinoic acid-inducible gene 1) -MAVS (mitochondrial antiviral-signaling protein) by host or viral dsRNA, or plasma membrane TLR4 by bacterial LPS (38, 61). The cell responsiveness to various DAMPs or PAMPs relies on the expression of pattern recognition receptors (PRRs). pDCs express high levels of TLR7 and TLR9, therefore pDC can rapidly sense self-DNA released upon injury then produce IFNα (13, 26, 39). While TLR4 and TLR8 are usually not expressed in pDC, these PRRs are highly expressed in classical DC or monocytes (62), making these cells highly responsive to bacterial LPS or self-RNA. In contrast to these myeloid derived immune cells, keratinocytes express high levels of TLR3 and MAVS, but not TLR4, 7, 8, or 9 (2, 30, 31). Therefore, keratinocytes rapidly produce IFNβ in response to dsRNA but not to TLR4, 7, 8, or 9 ligands (2). We have showed that, wounded keratinocytes upregulate the expression of antimicrobial peptide LL37, which then enables MAVS and TLR3 in keratinocytes to recognize dsRNA released from dying cells (2). By MAVS-dependent activation of TBK1 (TANK-Binding Kinase 1)-AKT (AKT serine/threonine kinase 1)-IRF3 (interferon regulatory factor 3) signaling pathway, keratinocytes produce and secrete IFNβ (2). Keratinocyte-derived IFNβ then promotes DC maturation and the subsequent T cell activation to facilitate the development of an autoimmune cutaneous inflammatory response (2). These results show that the cell type specific expression of pattern recognition receptors shape the unique and situation specific innate immune response of these cells.

## Role of dsRNA Signaling in Psoriasis Pathogenesis

Recent studies have suggested an important role for dsRNA in autoimmune initiation during psoriasis pathogenesis. Extracellular RNA complexes have been found in psoriatic skin (62), and dsRNA is also detected in the cytosol of wounded or psoriatic KCs while it is normally localized in the nucleus of KCs in normal skin (2). Recently, it has been shown that the dsRNA accumulation in psoriatic keratinocytes is associated with impaired A-to-I RNA editing activity, which is essential for post-translational modification of dsRNA and unwinding of dsRNA structures (63). In line with these observations, functional analysis of the psoriasis susceptible gene implicates the involvement of innate immune responses to dsRNA in disease progression (64).

Accumulating evidences have demonstrated that dsRNA can be released as a DAMP by damaged cells or a PAMP by invading viruses to initiate host immune activation. We and others have shown that dsRNA released by tissue damage activates and TLR3 and it is required for normal inflammation or skin regeneration (30, 65). Viral dsRNA or endogenous dsRNA (such as U1 RNA) released upon injury acts on keratinocytes through the mitochondrial MAVS pathway to produce IFNβ, leading to adaptive immune activation (2, 31, 66). Skin injury or infection strongly induces the expression of cathelicidin antimicrobial peptide expression in KCs (2, 67), and LL37 (the α-helical polypeptide derived from cathelicidin) forms pro-inflammatory nanocrystalline complex with dsRNA that potentiates pattern recognition receptor clustering and immune amplification (2, 68). Together these recent evidences have shown that dsRNA released upon infection or injury may synergize with the host antimicrobial peptide to initiate the autoimmune activation in psoriasis, and this dsRNA immune response in keratinocytes may explain the Koebner phenomenon and why viral infection and wounding triggers psoriasis.

## Targeted Therapies for Psoriasis: Current Status and Future Developments

Psoriasis is not just a T cell mediated disease. New biological drugs targeting the TNF/IL-23/IL-17 pathways have shown to be safe and efficacious in recent psoriasis clinical trials (9, 19, 20). However, potential problems including lack of long term efficacy and rapid regain of psoriasis upon drug removal (22, 23, 25) suggest that inhibiting T cell activation is only effective to alleviate the disease symptoms but it cannot cure the disease. If the initiating factors such as type 1 IFNs are still active, and these factors can quickly re-initiate the inflammatory cascade and reactivate pathogenic T cells upon withdrawal of the T cell targeted therapy. In addition, studies has suggested that cytokines including type 1 IFNs, TNF, and IL17, are interwoven, and each of these cytokines is the cornerstones of an inflammatory triangle that drives the development and maintenance of psoriasis (69). Targeting one of these cytokines may affect the others. For example, patents treated with TNF blocking agents sometimes develop paradoxical psoriasis and this is resulted from an overproduction of IFNα from pDCs upon TNF inhibition (70–72). Together, these clinical observations indicate that inhibiting activation of the innate immune system of KCs or pDCs that initiates the autoimmune cascade may be needed in addition to targeted T cell therapy to prevent reoccurrence of the disease upon drug withdrawal. Targeting MAVS orTLR3 in KC to prevent IFNβ production from the skin epidermis is of great potential for future targeted therapy development to treat psoriasis.

## Conclusion

Located at the skin surface, keratinocytes are constantly exposed to a variety of stimuli, and can secrete an array of cytokines, which function as secondary messengers connecting the innate immune with adaptive immune system. Type 1 IFNs, which are the essential mediator of autoimmunity and antivirus host defense, have emerged as an important initiating element in the immunopathology of autoimmune cascade and psoriasis. In contrast to dermal pDC which produce IFNα in response to ssRNA or DNA, keratinocytes express high levels of MAVS and TLR3 and therefore can sense self-dsRNA in the presence of antimicrobial peptide and to produce IFNβ. Production of Type 1 IFNs from these innate immune cells upon skin injury may explain the Koebner phenomenon, and this may also explain psoriasis triggered by other factors such as bacterial or viral infections as RNA or DNA released from pathogens will activate similar innate immune response. Future studies will be needed to develop targeted therapy to block the innate immune activation of these PRRs in keratinocytes, which may result in more sustainable interventions to treat psoriasis in addition to current T cell targeted therapies.

## Author Contributions

The author confirms being the sole contributor of this work and has approved it for publication.

### Conflict of Interest Statement

The author declares that the research was conducted in the absence of any commercial or financial relationships that could be construed as a potential conflict of interest.
